# Control beliefs as mediators between education and quality of life in patients with breast, prostate, colorectal, and lung cancer: a large register based study

**DOI:** 10.1186/s40359-024-01867-7

**Published:** 2024-07-09

**Authors:** Julia Roick, Peter Esser, Beate Hornemann, Jochen Ernst

**Affiliations:** 1 Department of Sport and Health Sciences, Chair for Social Determinants of Health, Technical University of Munich, Georg-Brauchle-Ring 60/62, 80992 Munich, Germany; 2 Department of Medical Psychology and Medical Sociology, University Medical Center Leipzig, Philipp-Rosenthal-Straße 55, 04103 Leipzig, Germany; 3 Department of Psychooncology, University Cancer Center (NCT/UCC), University Hospital Carl Gustav Carus, Dresden, Fetscherstrasse 74, 01307 Dresden, Germany

**Keywords:** Control beliefs, Quality of life, Cancer, Education, Mediation analyses

## Abstract

**Objective:**

Control beliefs have been found to influence adaption to a cancer diagnosis. This study explored interrelationships among education, control beliefs, and health-related quality of life (HRQoL) in patients with breast, prostate, colorectal, and lung cancer and tested weather control beliefs act as mediators.

**Methods:**

Six hundred and five patients with breast (*n* = 205), prostate (*n* = 205), colorectal (*n* = 124), and lung (*n* = 71) cancer from two German cancer registries answered standardized questionnaires. Response rate was 54%. HRQoL was assessed with the EORTC QLQ-C30 core questionnaire and control beliefs (internal, external, and fatalistic) were evaluated using the IPC-questionnaire. Education was measured on a scale ranging from 1 to 8. Data were analyzed using multiple mediation models.

**Results:**

There was a positive correlation between education and HRQoL. Internal beliefs were positive and external beliefs were negative correlated with HRQoL. Internal control beliefs mediated the relationship between education and global health-related quality of life (.299, CI .122, .531), physical functioning (.272, CI .110, .486), emotional functioning (.325, CI .120, .578), and pain (-.288, CI − .558, − .094). External and fatalistic control beliefs did not act as mediators.

**Conclusion:**

Patients with low education feel they have less control over their cancer disease and consequently a poorer health-related quality of life.

**Supplementary Information:**

The online version contains supplementary material available at 10.1186/s40359-024-01867-7.

## Introduction

In Germany, almost 500.000 people are diagnosed with cancer every year and the number is constantly rising [1]. Different reviews concluded that cancer patients suffer from elevated anxiety and depressive symptoms [2, 3]. Cancer not only affects the mental health of the patients but also their physical condition. In particular, persistent pain and fatigue affect the well-being of patients [4]. As a result, health-related quality of life (HRQoL) can be markedly impaired. HRQoL is a multidimensional construct consisting of objective parameters and the subjective evaluation by the person. In cancer research, the focus on HRQoL has become important in recent years due to success in long-term survival. HRQoL is now considered the most important treatment goal after survival. Coping with the disease is closely related to HRQoL [5]. Especially the cognitive assessment of the disease situation makes a decisive contribution to the patients’ well-being. For instance, a study with patients with head and neck cancer shows that causal attributions about causes of the disease and course of the symptoms as well as symptom perceptions are associated with HRQoL [6].In this context, the importance of different types of control beliefs as coping mechanisms were discussed. Control beliefs are described as personality traits in the social learning theory of Rotter [7]. Control beliefs are one aspect of general expectations. Different manifestations of control beliefs are considered as variables for explaining specific behavior and stress experiences. Internal control beliefs are present when a person assumes that events that occur are due to his or her own behavior [8]. If a person attributes events to the actions of others or assumes that they are determined by chance or fate, control beliefs are referred to as external. External control beliefs are further divided into social externality (influence by other persons) and fatalistic externality (fate or luck). Research focusing health-related control beliefs for specific diseases are inconclusive so far. For example, internal control beliefs are associated with faster progression in subsequent medical rehabilitation in patients after knee arthroplasty and with better disease adjustment after HIV infection [9, 10]. Contrary, external control beliefs were associated with better glucose metabolism in persons with type I diabetics at the beginning of the disease [11]. Findings on control beliefs in oncology research are highly dependent on the research topic. In the area of causal attributions, it has been shown that internal attributions of causes of the disease predominantly lead to a more depressive experience of the disease and, as a consequence, to maladaptive coping [5]. However, internal causal attributions are more likely to lead to changes in health behaviors to prevent disease recurrence [12, 13]. Focusing the perceived control over cancer, a meta-analysis shows that internal control beliefs are associated with better coping [14]. The higher the controllability, the less anxiety and depression patients report. Further, internal control beliefs are related to better HRQoL in patients with gastric, colorectal, and lung cancer [15]. In patients with head and neck cancer, control beliefs show only partial associations with HRQoL, depending on the age of the patients [16].

There is evidence that socioeconomic factors, like income or education, are associated with well-being for many diseases. Studies examining associations between education and HRQoL in cancer patients are inconclusive so far. One study found that education was associated with HRQoL only in older but not in younger patients [17]. Examining effects of education on HRQoL in oesophageal cancer patients, associations were only found for women, but not for men [18], however, in a sample of prostate cancer patients, those with low education reported worse HRQoL [19]. With regard to the timing during treatment, Simon and Wardle found disparities in anxious and depressive symptoms and general HRQoL to the disadvantage of cancer patients with low status only directly after diagnosis [20]. A study with mixed cancer patients found no associations between educational level and HRQoL up to six months after hospitalization [21]. Regarding control beliefs, previous research has shown that individuals with low education and in occupational positions with less prestige are more likely to believe that their health is determined by social or fatalistic externality. In a study with healthy persons and persons with neuromuscular diseases, associations with school education were only found for the subscale fatalistic externality in that way, that persons with lower education achieved higher values [22]. Although previous literature suggests that education is associated with both control beliefs and HRQoL, it remains unclear whether there is an indirect effect of education on HRQoL via control beliefs

## Current study

The current study explored whether control beliefs mediate the association between education and HRQoL in patients with breast, prostate, colorectal, and lung cancer.

The four most common diagnostic groups in Germany were selected in order to be able to use the diagnostic group from the sample as a meaningful control variable on the one hand and to be able to generate a sufficiently large total sample that met our inclusion criteria (time since diagnosis five years) on the other.

## Method

### Participants and procedure

**Fig. 1:**
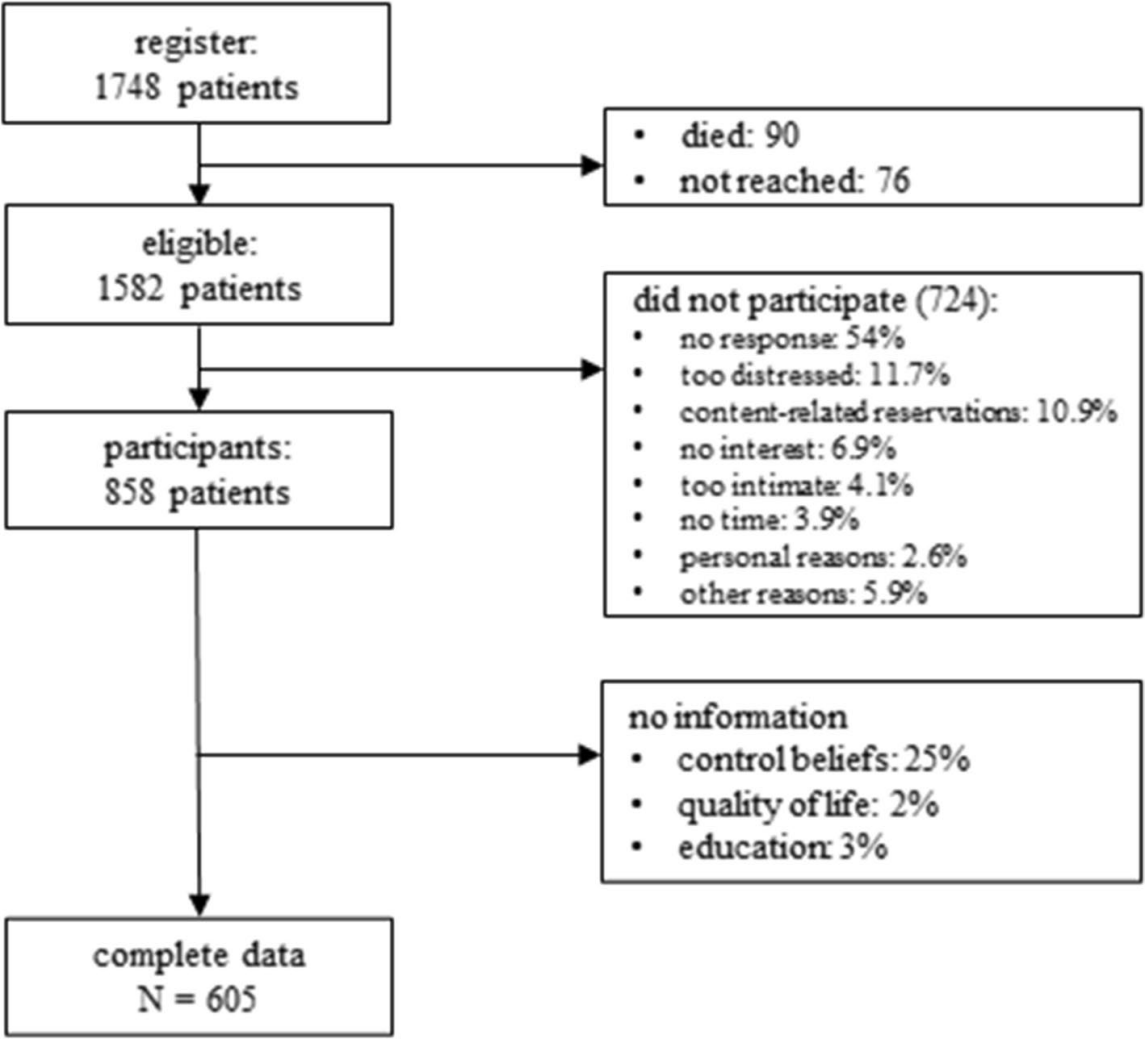
Flowchart of the sample

Data collection was carried out between May and September 2016 in two German cancer registries (cities of Leipzig and Dresden). Patients with breast, prostate, colorectal, or lung cancer who met the following inclusion criteria were eligible for the study: (I) age between 18 and 75 years, (II) new diagnosis or relapse, (III) time of diagnosis or relapse not more than 30 months ago, and (IV) written informed consent. The selection of study participants was stratified by cancer site to obtain equally sized groups despite differences in incidence rates. A total of 1748 patients were contacted by mail and asked to complete a standardized questionnaire. In case of no reply, up to two reminder letters were sent. Of 1748 patients contacted, 166 (9.4%) were already deceased or could not be reached, leaving 1582 eligible patients for this study (Fig. 1). Of those, 858 (54.2%) participated. This study was granted ethical approval by the Ethics Committee of the Medical Faculty of Leipzig (AZ 342-15-05102015) and the University of Dresden (EK 442102015).

### Instruments

#### Sociodemographic and medical data

Cancer diagnosis according to ICD-10 were taken from cancer registries. Tumor stage was classified according to the Union for International Cancer Control [UICC] classification system seventh edition [23]. Other sociodemographic and medical data were assessed via self-report.

Education was calculated using the Hoffmeyer-Zlotnik index [24] which is a combination of the highest level of school education and the highest level of vocational training. Based on the combination, patients have been assigned a value for education on a scale from 1 to 8 (e.g., post compulsory school and apprenticeship = 4).

#### Health-related quality of life

HRQoL was measured using the German version of the European Organisation for Research and Treatment of Cancer Quality of Life Core Questionnaire (EORTC QLQ-C30) [25]. The EORTC QLQ-C30 can be summarized into 5 functional scales (emotional, physical, social, role, and cognitive functioning) and 3 multi-item (fatigue, pain, and nausea and vomiting), and 6 single-item scales that assess cancer-related symptoms (dyspnoea, loss of appetite, insomnia, constipation and diarrhoea) and global HRQoL. Responses are given on a four-point Likert scale that ranges from 1 (“not at all”) to 4 (”very much”), with the exception of global HRQoL for which the rating scale ranges from 1 (“very poor”) to 7 (“excellent”). The scales were transformed into values between 0 and 100 according to the scoring manual [26]. Higher scores on the functional and global HRQoL scales and lower scores on the symptom scales indicate better HRQoL. Because a cancer diagnosis and its therapy affects a patients physical and mental health and can cause persistent pain even years after end of therapy, we focused on the scales global HRQoL (2 items, Cronbach’s Alpha = .93), physical functioning (5 items, α = .83), emotional functioning (4 items, α = .90), and pain (2 items, α = .8) in our analyses.

#### Control beliefs

General control beliefs were assessed using the German version of the IPC-questionnaire [27] which has been developed on the basis of Levenson's [28] IPC scales. The self-report instrument consists of 24 items that can be answered on a six-point Likert scale ranging from 1 (“very wrong”) to 6 (“very right”). The items constitute 3 subscales, each consisting of 8 items, with values ranging from 8 to 48. The internality scale (I-scale, α = .72, e.g., „ I can determine quite a lot of what happens in my life by myself.“) measures the perceived control over events that occur. The externality scale (P-scale, α = .73, e.g., „ Whether I have a car accident or not depends mainly on the other car drivers.“) determines the subjective feeling of powerlessness over events or the dependence on other persons. The fatalism scale (C-scale, α = .76, e.g., „ It is not good for me to plan far in advance, because fate often intervenes.“) captures the subjective feeling that events are dependent on luck or fate. Patients with high values in one of the scale have high internal, external, or fatalistic control beliefs.

### Statistical analyses

Pearson’s chi-square-test and Fisher's exact test were used to compare participants and non-participants regarding age, sex, tumor stage, and cancer site. In addition, study participants with and without information on their control beliefs were compared.

**Fig. 2:**
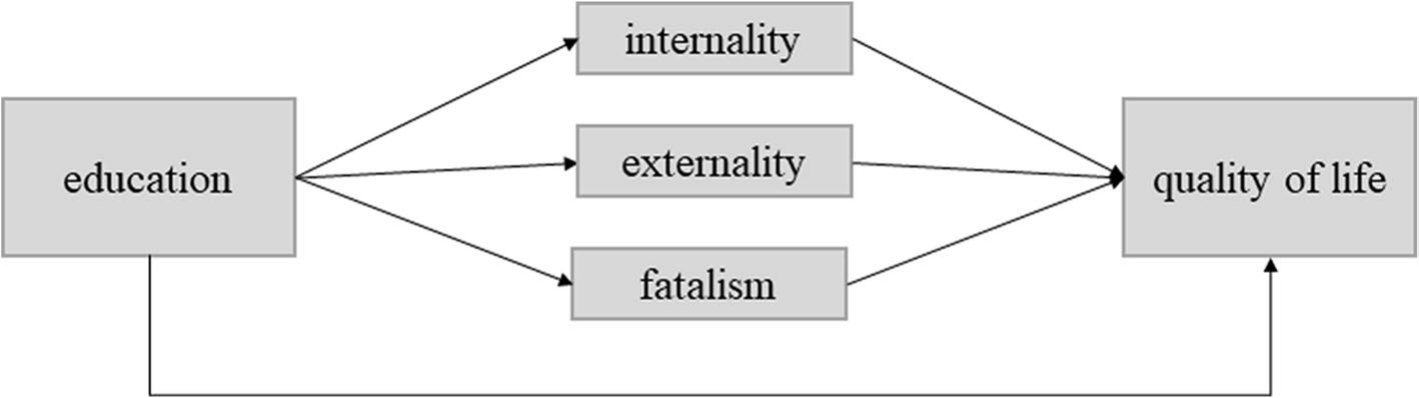
Theoretical model in which control beliefs mediate the relationship between education and health-related quality of life

To test associations between education, HRQoL, and control beliefs, bivariate correlations among the study variables were calculated in a first step. In a second step, multiple mediation analyses were calculated in order to estimate the overall indirect effect and the specific indirect effects [29]. Multiple mediation models test whether an independent variable (X) predicts an independent variable (Y) via a mediator variable (M). Our theoretical model is schematically depicted in Fig. 2. We assumed a direct effect of education on HRQoL and additional indirect effects via control beliefs. We calculated 4 models with HRQoL domains as dependent variables and education as independent variable. In all models, we added control beliefs (internal, external, fatalistic) simultaneously as potential mediators. Age, gender, and tumor site were added as potential confounders to the models. Data analyses were conducted using the IBM SPSS Statistics Version 25 and the macro PROCESS provided by Preacher and Hayes (2008). The significance of indirect effects was tested by bootstrap analyses with 5000 bootstrap samples. The dataset is made available in the figshare online repository [30].

## Results

### Sample characteristics

As can be seen from Fig. 1, the most frequent reasons for non-participation were patient felt too distressed (11.7%), content-related reservations (10.9%), and no interest (6.9%). Participants and non-participants did not differ with respect to sex (*p* = .66) and age (*p* = .75), but in terms of cancer site and stage of disease in that way that patients with breast and prostate cancer (*p* = .02) and with lower disease stage (*p* = .03) participated more often. Of the 858 participants, 16 (1.9%) did not provide any information on HRQoL, 211 (24.6%) on control beliefs, and further 26 (3%) did not provide their educational level, leaving 605 patients for our analyses. Patients with information on control beliefs did not differ from those without information in disease stage (*p* = .82). Nevertheless, participants providing data on control beliefs are more likely to be male (*p* = .04) and younger (*M* = 59.4) compared to those without data (*M* = 64.1, *p* < .01). In addition, patients with available data on control beliefs are higher educated (*M*_NoData_ = 4.7, *M*_WithData_ = 5.4, *p* < .01) and are less likely to be lung cancer patients compared to prostate cancer patients (*p* < .01) and colon cancer patients (*p* = .02).

At the time of the survey, the 605 participants included were 59.4 years (*SD* = 9.5), ranging from 23 to 73 years (Table 1). More than half of the patients (56.9%) are male. The majority of the study participants had completed high school (44.3%) and an apprenticeship (47.4%). Of the respondents, 33.9% received a diagnosis of breast cancer, 33.9% prostate cancer, 20.5% colorectal cancer, and 11.7% lung cancer. The mean time since diagnosis was 1.8 years (*SD* = 1.4).

**Table 1:** Sample characteristics

		N	%
**Age**	Mean [SD] in years	59.4 [9.5]	
**Sex**	Male	344	56.9
**School education**	Compulsory	68	11.2
	Post-compulsory	259	42.8
	High school	268	44.3
	None	10	1.7
**Vocational training**	Apprenticeship	287	47.4
	Higher	109	18.0
	University	155	25.6
	None	9	1.5
	Other	45	7.4
**Tumor stage**^a^	0/I	122	20.2
	II	50	8.3
	III	56	9.3
	IV	32	5.3
	No information	345	57.0
**Cancer site**	Breast	205	33.9
	Prostate	205	33.9
	Colorectal	124	20.5
	Lung	71	11.7

^a^Data on tumor stage is only available for the study center Leipzig

### Bivariate associations between health-related quality of life, control beliefs, and education

Means, standard deviations, and results of the correlation analyses are presented in Table 2. There is a positive correlation between internal control beliefs and HRQoL. Also, education is positively correlated with HRQoL and internal control beliefs. There was no relationship between education and external control beliefs.

**Table 2:** Bivariate correlations and descriptive information of all study variables (*N* = 605)

	GQoL	PF	EF	Pain	ICB	ECB	FCB	Edu
Interrelationships								
1. GQoL^a^	-	-	-	-	-	-	-	-
2. PF^a^	**.63**	-	-	-	-	-	-	-
3. EF^a^	**.61**	**.51**	-	-	-	-	-	-
4. Pain^b^	**− .61**	**− .63**	**− .57**	-	-	-	-	-
5. ICB^c^	**.21**	**.21**	**.21**	**− .17**	-	-	-	-
6. ECB^c^	**− .22**	**− .21**	**− .37**	**.15**	− .03	-	-	-
7. FCB^c^	**− .29**	**− .28**	**− .40**	**.23**	− .07	**.71**	-	-
8. Edu	**.14**	**.21**	**.09**	**− .15**	**.17**	.02	− .03	-
Descriptive Information								
* M*	65.26	77.29	68.57	29.20	35.96	21.02	21.83	5.57
SD	21.37	22.26	27.00	29.64	5.18	5.43	5.76	1.94

Coefficients in bold show a significant correlation

*GQoL* Global quality of life, *PF* Physical functioning, *EF* Emotional functioning, *ICB* Internal control beliefs, *ECB* External control beliefs, *FCB* Fatalistic control beliefs, *Edu* education

^a^Higher scores reflect better quality of life (scale ranges from 0-100)

^b^Higer scores reflect higher symptom burden (scale ranges from 0-100)

^c^Higher scores reflect a higher manifestation in that control belief (scales range from 8-48)

### Indirect effects of education on quality of life through control beliefs

Multiple mediation analyses (Table 3) revealed a significant total indirect effect of the set of control beliefs (sum of internal, external, and fatalistic control beliefs) on global HRQoL (.410 (SE [standard error] .159), CI [confidence interval] .108, .740), physical functioning (.381 (.151), CI .103, .694), and pain (-.412 (.176), CI − .779, − .081), but not on emotional functioning (.433 (.245), CI − .019, .925). Regarding specific (unique) indirect effects of the dimensions of control beliefs, results show that controlling for all other control beliefs, only internal beliefs significantly mediate the relationship between education and global HRQoL (.299 (.105), CI .122, .531), physical functioning (.272 (.098), CI .110, .486), emotional functioning (.325 (.117), CI .120, .578), and pain (-.288 (.118), CI − .558, − .094). External control beliefs and fatalistic control beliefs did not show an indirect effect on all HRQoL domains and pain. Adding control beliefs as mediators to the models, there was still a direct effect of education on global HRQoL (.922 (.389), CI .159, 1.686), physical functioning (1.713 (.395), CI .489, .937), and pain (-1.323 (.554), CI -2.411, − .235), but not on emotional functioning (.618 (.469), CI − .303, 1.538), indicating that the effect of education on emotional functioning is fully mediated by internal control beliefs. Information about direct effects for all models are presented as supplemental material.

**Table 3:** Multiple mediation analysis testing effects of education on health-related quality of life via control beliefs

		Bootstrap analyses with 5000 bootstrap samples		
**Health-related quality of life domain**	**Mediator (control beliefs)**	Indirect effect (SE)	Lower 95% CI	Upper 95% CI
Global quality of life	Total indirect effect	.410 (.159)	.108	.740
	Internal	.299 (.105)	.122	.531
	External	− .002 (.026)	− .060	.053
	Fatalistic	.113 (.106)	− .085	.346
Physical functioning	Total indirect effect	.381 (.151)	.103	.694
	Internal	.272 (.098)	.110	.486
	External	− .002 (.026)	− .059	.054
	Fatalistic	.111 (.107)	− .083	.340
Emotional functioning	Total indirect effect	.433 (.245)	− .019	.925
	Internal	.325 (.117)	.120	.578
	External	− .019 (.120)	− .248	.192
	Fatalistic	.127 (.119)	− .086	.387
Pain	Total indirect effect	− .412 (.176)	− .779	− .081
	Internal	− .288 (.118)	− .558	− .094
	External	.001 (.033)	− .069	.076
	Fatalistic	− .125 (.123)	− .381	.103

Analyses controlled for age, sex, and tumor site

*CI* confidence interval

## Discussion

The present cross-sectional study investigated control beliefs as mediators between education and HRQoL in patients with breast, prostate, colorectal, and lung cancer. Our findings suggest that internal control beliefs partly mediate the effect of education on global HRQoL, physical functioning, and pain and fully mediate the effect on emotional functioning.

Our findings confirm past research among non-oncological populations showing that control beliefs are associated with education [22, 31, 32]. While these studies primarily found associations between external control beliefs and education, in our study with cancer patients only internal control beliefs were related to education. However, the studies cited used instruments that assessed health-related and not general control beliefs, as we did. Besides, education is crucial for acquiring coping skills and problem solving competencies [31] so that cancer patients with high education are more likely to think that they can actively shape their own lives. This in turn can result in a better HRQoL. Previous studies focusing on HRQoL and control beliefs in cancer patients are inconclusive. Whereas in a meta-analysis internal control was associated with better well-being [14], others report a maladaptive disease adjustment [5]. These differences may possibly have resulted from different perceived controllability depending on disease severity and tumor entity.

Our results also replicate previous studies that found higher education to be associated with better HRQoL [19, 33]. Two possible explanations are conceivable for the occurrence of educational inequalities in HRQoL. First, it was found that individuals with low education have information deficits about their disease and about possible support in aftercare, which may be negatively related to coping with the disease [34]. Second, patients with lower education may have greater worries and worse recovery, because they have a lower income and thus concerns about financial burden are stronger or they do not have the opportunity to pause work for subsequent rehabilitation. Studies with cancer patients show that people with low social status are less likely to use rehabilitation services [35].

The mediating effect of internal control beliefs in the relationship between education and HRQoL follows previous research [36]. Because in the present study the relationship between education and HRQoL is only partially mediated by internal control beliefs for most of the domains, education and control beliefs each appear to have an independent influence on HRQoL.

### Strengths and limitations

The present study contributes to a better understanding of the interplay between education, HRQoL, and control beliefs and thus provides a further explanation for existing inequalities in the HRQoL of cancer patients. Furthermore, we used a large sample based on two registers so that we can provide robust results in a research field which has rarely been investigated before. Another strength is that central medical data was collected via cancer registries and are therefore very valid. Nevertheless, some limitations need to be considered when interpreting our results. First, we used a cross-sectional design which is why the results presented cannot be interpreted causally. Second, the sample was limited to the four most common cancer sites, so the reported results may not be generalized to all cancer types. Also, participants and non-participants differ in terms of tumor entity and disease severity what could have biased the sample. Another point is that those who provided information on their control beliefs were more likely to be male, younger, and higher educated which limits the generalizability of the results for the whole sample. One reason for the high number of missing responses on control beliefs may be due to some of the questions themselves. In particular, questions that are very close to job-related content were not answered (e.g.: “Although I am capable of doing this, I am rarely given management tasks.” or “Whether I become a group leader or not depends above all on being in the right place at the right time.”). It is possible that the participants were not working and therefore had no connection to the questions. This also explains why it was mainly younger people who gave information on their control beliefs. Finally, control beliefs were assessed by means of a questionnaire, which exclusively depicts general control beliefs of the persons and does not include disease-specific control beliefs.

### Implications

The present work suggests that cancer patients with a low level of education have a reduced HRQoL. In treatment and aftercare, they are a vulnerable group of patients who need special support. Since educational attainment is easy to assess, this could be an ecological way to quickly identify individuals in need of support in clinical practice and to initiate appropriate interventions. For example, patients with low education should receive more detailed and easy understandable information about their disease from their physician to reduce uncertainty and strengthen internal control beliefs. Furthermore, promoting health literacy can improve disease-related coping skills and thus strengthen internal control beliefs in patients. Subsequent studies should be longitudinal to better track changes in control beliefs and resulting potential changes in HRQoL in cancer patients. Also, differently perceived controllability with regard to disease severity and tumor entity would be an interesting point to investigate. Furthermore, future research should include multiple cancer sites to explore control beliefs in cancers with unfavorable courses (e.g., pancreatic cancer). This could indicate to what extent the severity of a disease affects control beliefs. To assess control beliefs, another instrument should possibly be used that relates content less to the work context. This could prevent non-working people (e.g. retired, unemployed) from omitting the relevant questions.

### Supplementary Information

Supplementary Material 1.

## Data Availability

The dataset is made available in the figshare online repository. https://doi.org/10.6084/m9.figshare.19078064.v1
