# Supplementary material for: Control beliefs as mediators between education and quality of life in patients with breast, prostate, colorectal, and lung cancer: a large register based study
Source: BMC Psychol. 2024 Jul 9;12:382. doi: 10.1186/s40359-024-01867-7 (PMC11232264; doi:10.1186/s40359-024-01867-7)
Supplement: Supplementary file 1 — Supplementary Material 1. [file 40359_2024_1867_MOESM1_ESM.pptx]

## Slide 1
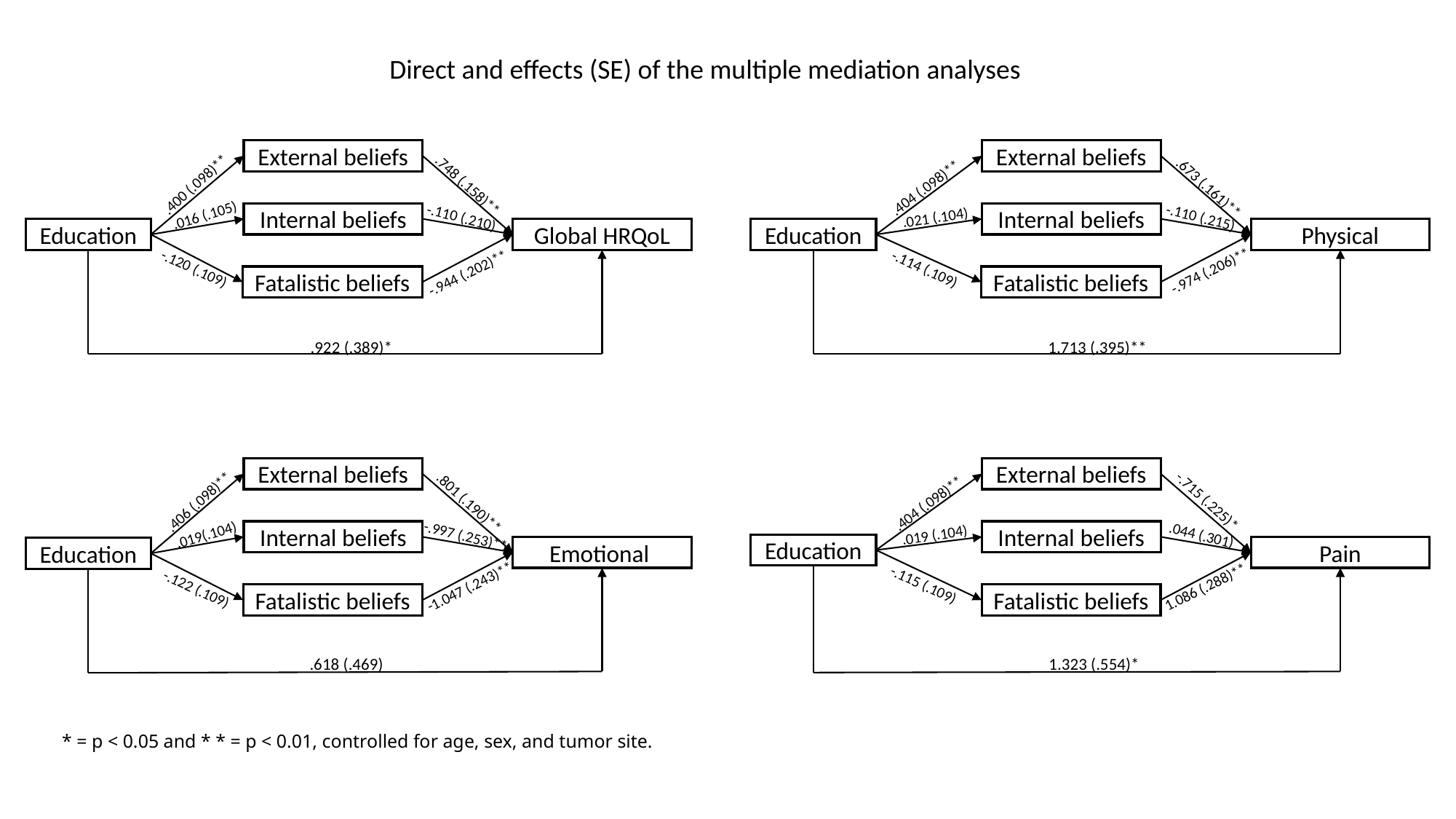

Direct and effects (SE) of the multiple mediation analyses
External beliefs
External beliefs
.400 (.098)**
.748 (.158)**
.404 (.098)**
.673 (.161)**
.016 (.105)
.021 (.104)
-.110 (.210)
-.110 (.215)
Internal beliefs
Internal beliefs
Global HRQoL
Physical
Education
Education
-.120 (.109)
-.114 (.109)
-.974 (.206)**
-.944 (.202)**
Fatalistic beliefs
Fatalistic beliefs
.922 (.389)*
1.713 (.395)**
External beliefs
External beliefs
-.715 (.225)*
.406 (.098)**
.801 (.190)**
.404 (.098)**
.019(.104)
.019 (.104)
.044 (.301)
Internal beliefs
Internal beliefs
-.997 (.253)**
Education
Emotional
Pain
Education
-.115 (.109)
-1.047 (.243)**
1.086 (.288)**
-.122 (.109)
Fatalistic beliefs
Fatalistic beliefs
.618 (.469)
1.323 (.554)*
* = p < 0.05 and * * = p < 0.01, controlled for age, sex, and tumor site.
